# The ATM- and ATR-related SCD domain is over-represented in proteins involved in nervous system development

**DOI:** 10.1038/srep19050

**Published:** 2016-01-08

**Authors:** Lukas Cara, Medina Baitemirova, Jack Follis, Maia Larios-Sanz, Albert Ribes-Zamora

**Affiliations:** 1 Department of Biology, University of St. Thomas, Houston, TX, USA; 2 Department of Mathematics, Computer Science and Cooperative Engineering, University of St. Thomas, Houston, TX, USA

## Abstract

ATM and ATR are cellular kinases with a well-characterized role in the DNA-damage response. Although the complete set of ATM/ATR targets is unknown, they often contain clusters of S/TQ motifs that constitute an SCD domain. In this study, we identified putative ATM/ATR targets that have a conserved SCD domain across vertebrates. Using this approach, we have identified novel putative ATM/ATR targets in pathways known to be under direct control of these kinases. Our analysis has also unveiled significant enrichment of SCD-containing proteins in cellular pathways, such as vesicle trafficking and actin cytoskeleton, where a regulating role for ATM/ATR is either unknown or poorly understood, hinting at a much broader and overarching role for these kinases in the cell. Of particular note is the overrepresentation of conserved SCD-containing proteins involved in pathways related to neural development. This finding suggests that ATM/ATR could be directly involved in controlling this process, which may be linked to the adverse neurological effects observed in patients with mutations in ATM.

Ataxia telangiectasia (A-T) is a disease characterized by a progressive cerebellar ataxia accompanied by immunodeficiency and increased risk of cancer^1^,^2^,^3^. A-T patients bear mutations in the kinase ATM (ataxia telangiectasia mutated), that along with ATR (ATM and Rad3-related), coordinates the action of multiple pathways during the DNA damage response (DDR)^4^,^5^,^6^. When activated, ATM and ATR phosphorylate a panoply of targets that halt the cell cycle, activate DNA repair pathways, and trigger programmed cell death in case of non-repairable damage, among other pathways^7^,^8^,^9^. While ATM and ATR were originally placed at the top of a series of cascade events with a limited number of direct targets, an increasing number of identified putative targets support a more direct role for these kinases in controlling downstream effectors^10^,^11^,^12^,^13^. There is also growing evidence that ATM and ATR have a role in several pathways outside the DDR, including in the signaling response to hypoxia and hyperthermia, the insulin signaling pathway and mitochondria physiology^10^,^11^,^12^. While the role of ATM in DNA repair activation and maintenance of genome stability is likely to explain the immunodeficiency and increased cancer risk observed in A-T patients, the reasons for the neuropathological effects remain unclear and the possible ATM targets responsible for this phenotype have not yet been identified^14^,^15^,^16^,^17^,^18^.

Identifying a consensus recognition site on ATM and ATR targets has been problematic. ATM and ATR have a strong preference for phosphorylating serines and threonines that are followed by glutamine (S/TQ), and more than half of the better characterized ATM targets contain an SCD domain, which is defined as the presence of at least 3 S/TQ sites in a stretch of 100 amino acids^19^,^20^. The SCD domain is also found in more than half of the 686 proteins identified as possible ATM/ATR targets in a high throughput screen of human proteins that are phosphorylated in response to DNA damage^21^. Studies using a more stringent SCD definition (3 S/TQ in 50 amino acids) found that this domain is present 4 times more abundantly in the yeast proteome than would be expected by random chance alone^22^. Furthermore, ontology analysis also revealed that SCD proteins do not distribute randomly within the yeast proteome, but, instead, are over-represented in pathways known to be under ATM/ATR control^22^. Overall these data suggest that identifying SCD-containing proteins could help identify novel ATM or ATR targets, further enhancing our understanding of the role of these kinases in the cell.

One of the major caveats of using the presence of an SCD as a predictive tool for the identification of ATM/ATR targets is the possibility of the stochastic generation of an SCD within any given protein sequence. To minimize this problem, researchers have used more stringent SCD definitions or focused on pathways where SCD proteins are over-represented rather than looking at individual ATM/ATR targets^20^,^22^. In this study, we used the evolutionary conservation of SCDs across vertebrates as a filter to reduce background noise during proteomic SCD searches. The presence of an SCD in orthologs from different species increases the likelihood that it is a biologically relevant feature of the protein. Using this strategy, we identified 842 proteins whose homologs in human, mouse and zebrafish all contain at least one SCD in their sequence. Ontology analysis of these proteins revealed an over-representation of SCD-containing proteins that are involved in several pathways including nervous system development, suggesting that ATM and ATR may play bigger roles in this pathway than previously recognized and may be linked to the neurological defects observed in A-T patients.

## Results

We used *SCDFinder*^23^ to perform static database searches in the human, mouse and zebrafish proteomes, looking for proteins containing at least one SCD within their sequences. The results from each search were later cross-referenced using *Homologene* and we identified 842 SCD-containing human proteins whose homologs in mice and zebrafish also contain an SCD (see Supplementary Table S1). This group of proteins significantly overlaps with both a list of 81 well-known ATM targets (*p* < 10^−9^) and a list of 686 human proteins that contain phosphorylated S/TQ motifs identified in a large-scale proteomic analysis (*p* < 10^−61^)^21^.

**Figure 1:**
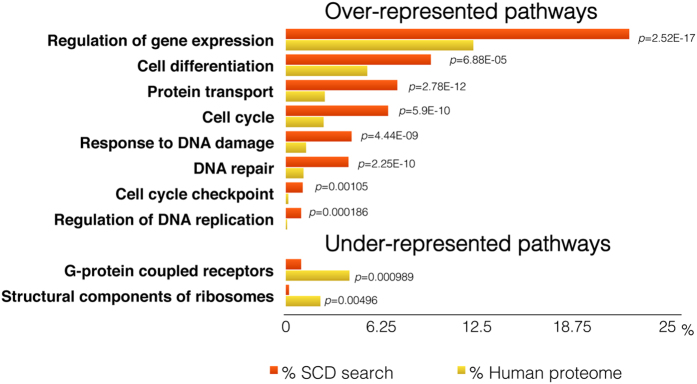
Selection of pathways with over-representation and under-representation of SCD-containing proteins. The percentage of proteins in our search that belong to each indicated pathway is represented by a red bar. Yellow bars indicate the percentage of proteins that belong to each pathway in the human proteome. The calculated *p* value for each pathway is shown.

Our ontology analysis indicates that these SCD-containing proteins do not distribute randomly in the human proteome. Rather, they are significantly over-represented in pathways known to be under ATM and ATR control, such as gene regulation (*p* < 10^−16^), cell cycle control (*p* < 10^−9^), DNA repair (*p* < 10^−9^) and regulation of DNA replication (*p* < 10^−3^) among others (Fig. 1). Importantly, proteins that play a role in the response to DNA damage are highly over-represented (*p* < 10^−8^), and proteins involved in pathways where ATM or ATR are not expected to play a role, like ribosomal assembly or G-protein couple receptors, are significantly under-represented in our analysis (*p* < 10^−3^ and *p* < 10^−2^ respectively), validating our approach (Fig. 1).

**Figure 2:**
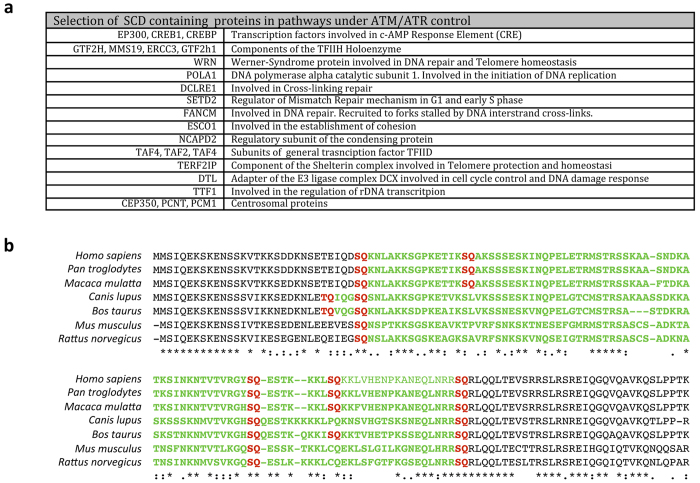
(**a**) Selection of novel putative ATM/ATR targets belonging to pathways known to be under ATM/ATR control. (**b**) Partial alignment of ESCO1 sequences showing SCD conservation across vertebrate species (excerpt of sequence from human, chimp, macaque, dog, cow, mouse, and rat homologs).

We also identified a selection of proteins that are not known to be phosphorylated by ATM or ATR but play a role in DDR-related pathways and contain an SCD that is consistently found across vertebrates (Fig. 2a). For instance, ESCO1 contains a conserved SCD and is involved in chromosome cohesion, a process that includes well-established ATM targets like SMC1^24^ (Fig. 2b). Also noteworthy is the presence of SCD domains in three important transcription factors that function through the DNA cAMP response element (CRE): CREB1, EP300 and CREBBP (Fig. 2a). This finding suggests a role for ATM or ATR in controlling transcription through this widespread regulatory element. Consistent with this is the fact that CREBBP was found to be phosphorylated in a high-throughput experiment that identified proteins with phosphorylated S/TQ motifs in the presence of DNA damage^21^. Other SCD-containing proteins identified in this study that are involved in transcription regulation include 3 subunits of TFIID and 4 out of the 8 subunits of the TFIIH holoenzyme (Fig. 2a).

We also identified several SCD-containing proteins that play key roles in DNA repair and that are mutated in patients suffering from Werner Syndrome (WRN) and Fanconi Anemia (FANCM) (Fig. 2a). In addition, the catalytic subunit of DNA polymerase alpha that is in charge of extending RNA primers during the initiation of DNA replication (POLA1) also contains an SCD that is found across vertebrates (Fig. 2a). Interestingly, DNA polymerase epsilon, which extends the initial product from DNA polymerase alpha, also contains an SCD across vertebrates, and was found to be phosphorylated at S/TQ sites following DNA damage^21^. Our approach also helped us identify several SCD-containing proteins involved in the microtubule cytoskeleton, which could be linked to the previously documented involvement of ATM in the mitotic spindle assembly checkpoint (SAC)^25^,^26^. These include several kinesins (KIF15 and KIF11), centromere proteins (CENPJ and CENPF) and key regulators of the mitotic spindle like NUMA1 and SPAG5. Our analysis also identified several centrosomal proteins (CEP350, PCNT and PCM1), which is interesting given the fact that the centrosome is a known location for ATM^27^,^28^ (Fig. 2a). Other noteworthy putative targets identified in pathways known to be controlled by ATM/ATR include a telomeric Shelterin component (TERF2IP), a cross-linking repair protein (DCLRE1a), the regulatory component of condensin (NCAPD2) and a regulator of rDNA transcription (TTF1) (Fig. 2a).

**Figure 3:**
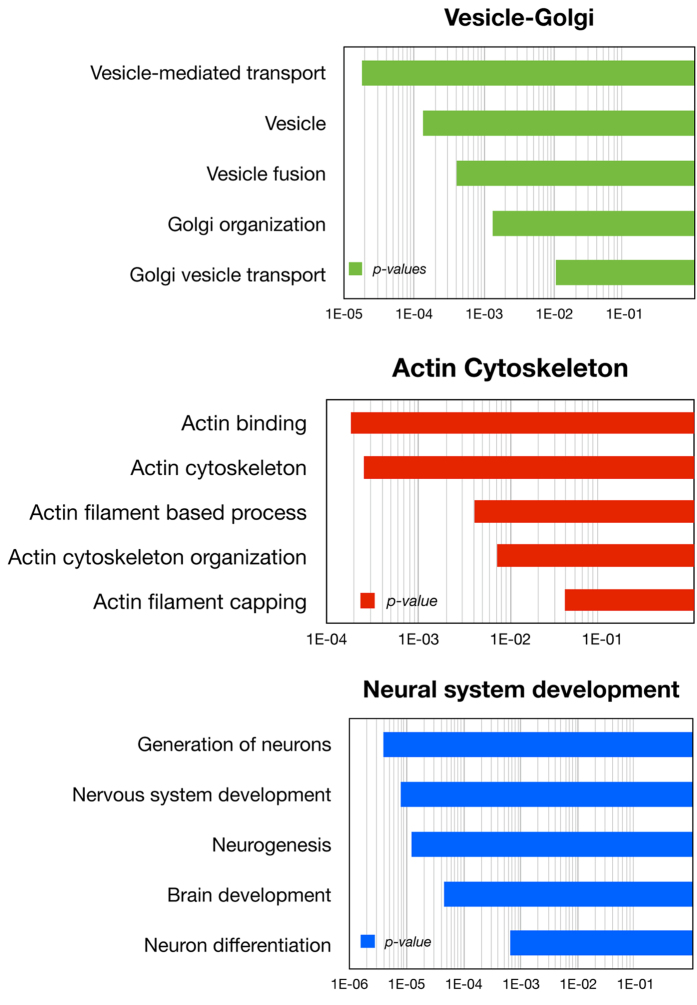
Pathways involved in vesicle-Golgi trafficking, actin cytoskeleton and neural system development that contain an over-representation of SCD-containing proteins. The calculated p value for each pathway is represented in bar graphs.

In this study, we have also detected a significant over-representation of proteins involved in pathways where the role of ATM or ATR is poorly understood, including those related to actin cytoskeleton and vesicle-Golgi trafficking (Fig. 3). These results are consistent with previously reported enrichments of SCD-containing proteins in these pathways in the yeast proteome^22^ and in the neural SQ phosphoproteome^29^. With respect to the vesicle-Golgi trafficking, we not only found novel putative ATM targets that are involved in Golgi maintenance, like COG7, USO1, GOLGA5, GOLGA2, GOLGB1 and GCC1, but we also identified proteins involved in the formation of the Clathrin-associated adaptor protein complex (AP1G1, AP2M1 and AP4M1), retrograde transport (VPS52 and COPB2), components of the COPII coat (SEC24B, SEC24D and SEC31B), SNARE proteins (STX17, STX4 and STX6) and the ESCRT-II complex (VPS36) among others. These results suggest that ATM could be more involved than anticipated in a wide variety of processes relating to vesicle trafficking.

Importantly, our analysis revealed several SCD-containing proteins that mediate the anchoring of vesicles and cisterns to the actin cytoskeleton through the ARF1/ARF3 system. Examples include ARFGEF1, CKAP4, HERC1 and HIP1R, as well as several myosins (MYH6, MYO3A, MYO5C and MYH7), Spectrin components (SPTBN5 and SPTBN1), members of the calponin complex (CNN1, CNN2 and CNN3) and RHOU. Intriguingly, ROCK2 kinase, a well know regulator of actin cytoskeleton that is also involved in centrosome duplication^30^, contains an SCD that is found across vertebrates and has been found to be phosphorylated at S/TQ sites following DNA damage^21^.

**Figure 4:**
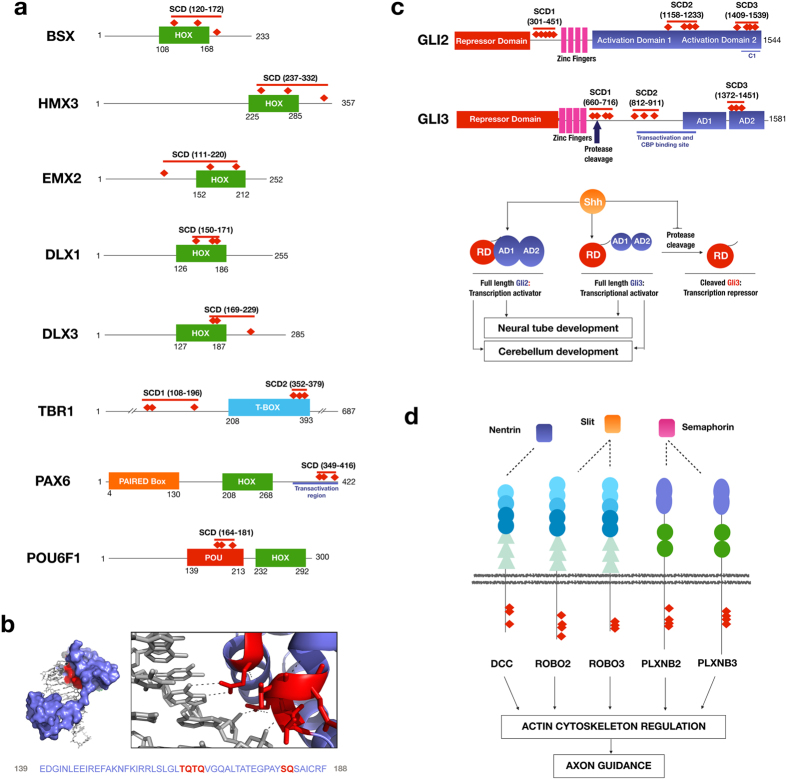
Miscellany of SCD-containing proteins involved in nervous system development that are putative ATM targets. (**a**) Examples of transcription factors that regulate different processes during neural development. The position of each SCD with respect to the DNA-binding domains is indicated and S/TQ motifs within SCDs are represented as red diamonds. (**b**) X-ray crystal structure diagram of the POU domain in the human transcription factor POU6F1 (PDB ID: 3D1N)^57^. The S/TQ motifs within the SCD are represented in red in the structure and in the partial sequence below (amino acid residues 139–188). The panel on the right shows different polar contacts between residues on the SCD and nitrogenous bases in the protein’s cognate DNA. (**c**) Top panel. Localization of SCDs within the transcription factors GLI2 and GLI3. Bottom panel. Current model for the role of GLI3 protease cleavage in the regulation of neurodevelopment. (**d**) SCD localization on the cytoplasmic side of transmembrane receptors involved in axon guidance on the growth cone of an axon.

A very interesting finding in this study is the highly significant enrichment of proteins with ontology terms related to neural development (Fig. 3). These proteins include several transcription factors that contain an SCD either within their DNA-binding domains or in their transactivation domain (Fig. 4a,c). Examples of these novel ATM target candidates include BSX, a brain-specific homeobox protein, DLX1, involved in forebrain development, TBR1, a T-box family transcription factor with important function in cortical development, and POU6F1, a transcription factor involved in pituitary development. In the case of POU6F1 for example, available structural data indicates that the conserved S/TQ motifs within the SCD are clustered on the surface of the protein and are directly involved in binding DNA through base interactions (Fig. 4b).

Other examples of SCD-containing proteins related to neurogenesis found in our study are EMX2 and PAX6, two important transcription factors involved in the specification and differentiation of cortical area patterns in the neocortex^31^,^32^. Additionally, GLI2 and GLI3 two major SCD-containing transcription factors under control of the sonic hedgehog pathway that drive spinal-cord patterning and cerebellum development^33^,^34^, also contain an SCD consistently found across vertebrates.

In addition to the transcription factors described above, we also found SCD domains within the cytoplasmic portions of receptors for every type of signal controlling axon guidance, a crucial process during neuron development and differentiation (DCC, ROBO2/3 and PLXNB2/3), and in cytoskeleton-related proteins with major roles during nervous system development (NES, CTTNBP2, NF1 and MAP1B), among others (Fig. 4d). The extent of putative targets in these pathways, as well as the presence of SCD domains in key regulatory and functional regions further points to a more direct and much broader role for ATM and ATR in the control of cellular processes than previously recognized.

## Discussion

While high-throughput studies have been instrumental in uncovering hundreds of novel possible ATM and ATR targets, these approaches are highly dependent on the experimental conditions of each study^21^,^35^,^36^,^37^. An ATM or ATR target that is phosphorylated only under certain conditions such as in a specific cell type, during a narrow range of time in development or in the presence of certain stimuli, will be missed by experiments that fail to reproduce these conditions. Bioinformatic approaches offer an alternative method to identify putative ATM and ATR targets in a way that is non-biased because they do not depend on specific experimental conditions. Using SCD*Finder*, we have identified 842 proteins that have at least one SCD domain in their sequence and that is present across vertebrates. The significant overlap of our list with known ATM and ATR targets and the high over-representation of proteins involved in pathways known to be controlled by these two kinases indicate that combining evolutionary data with SCD searches is an effective way to identify new putative targets under ATM and ATR control. Using this approach, we have identified several SCD-containing proteins that have known functions in ATM and ATR controlled pathways but whose status as direct targets for these kinases has not been established experimentally (Fig. 2).

While the presence of an SCD in a single protein could be the result of a stochastic event, its conserved presence in homologs from other species significantly reduces this possibility. Similarly, the presence of multiple SCD-containing proteins within a single pathway and across species is less likely to be due to chance. As illustrated in Fig. 1, the concentration of SCD proteins within a specific pathway may be an indication of possible ATM/ATR regulation. We found an overrepresentation of SCD-containing proteins in the actin cytoskeleton and in vesicle-Golgi trafficking, two pathways in the cytoplasm where the role for ATM is poorly understood. ATM has been found localized in cytoplasmic vesicles and in the Golgi apparatus forming a complex with β-COPI coatomer protein, but its function in this location remains unknown^38^,^39^. The presence of multiple SCD-containing proteins spread throughout different vesicle trafficking related pathways suggests a more widespread involvement for ATM and ATR in controlling vesicle trafficking than has been characterized to date. Our results also suggest broad and wide-ranging functions for ATM or ATR in regulating the actin cytoskeleton, a role that would be consistent with the cytoskeleton defects observed in cells derived from A-T patients^40^.

The role of ATM and ATR kinases in the neural system and their possible targets are starting to be elucidated. ATM has been found in the nucleus and cytoplasm of rat neuron cells and in neuron-like human cell lines^29^,^41^. Furthermore, ATM has been found active in the cytoplasm of rat neuron cells and bound to synaptic proteins like VAMP2 and Synapsin-I in mouse brain cells as well as to β-NAP, a neuron specific protein involved in vesicle transport^29^,^38^,^42^. Studies with neuron-like human cell lines have also shown ATM to be present in the cytoplasm and nucleus in both differentiated and non-differentiated cells^41^. Our finding of the over-representation of proteins associated with neural development that have an SCD present across vertebrates suggests that this may also be a pathway under direct ATM/ATR control. We have identified SCDs that are consistently found across vertebrates in several major transcription factors that control key processes during nervous system development, including cortical regionalization and area specification, cerebellar foliation and development, and the assembly of motor circuits and patterning in the spinal cord. These SCDs are often localized in DNA binding domains, activation domains (see Fig. 4) or other regulatory important regions like protease cleavage sites, as is the case, for example, of GLI3, whose transcription activity changes from activation to repression upon a protease cleavage event that occurs within the context of an SCD^43^ (see Fig. 4c). The presence of SCDs that are consistently found across vertebrates in the important regulatory regions of these proteins further suggests a role for ATM/ATR in regulating their function. These findings are also consistent with previously documented instances of SCDs in the transactivation domains of other transcription factors important in development, including Sp1^44^ and ASCIZ^45^.

Several key receptors that guide axon guidance during development also contain SCDs within their cytoplasmic region. These receptors react to guidance cues such as Semaphorins, Netrins and Slit proteins and elicit actin cytoskeleton rearrangements that drive axon attraction or repulsion during axon guidance^46^. This suggests that ATM/ATR could be involved in modulating the downstream response of these receptors during neural development. A possible role for ATM/ATR in actin cytoskeleton regulation could also influence cytoskeleton rearrangements during axon guidance. It is also possible that the role of ATM in vesicle trafficking and in the actin and microtubule cytoskeleton may be related to the transport of neurotransmitter vesicles to synapses or to neuron migration during differentiation.

A role for ATM in regulating different events during nervous system development may be connected to the neurodegeneration observed in A-T patients. It is not uncommon to find proteins with roles in neural development that are also implicated in neurodegeneration diseases, either because developmental defects set the stage for future neurodegeneration events or because, in addition to nervous system development, the protein also has a function in adults cells^47^,^48^. Examples of this are the Notch and Wnt pathways, which are essential during neurodevelopment but have also been implicated in neurodegenerative diseases such as Alzheimer’s disease or Parkinson disease, respectively^49^,^50^,^51^,^52^. Similarly, the neurodevelopmental disorder Fragile-X Syndrome is caused by silencing of FMRP, a protein that has also been implicated in neurodegeneration^53^. Several SCD-proteins listed in our study play a role in neurodevelopment and are also active in differentiated adult cells. This is especially accentuated in proteins involved in the cytoskeleton or involved in dendritic spine formation in differentiated neurons. For instance CTTNBP2 is a neuron-specific F-actin associated SCD-protein that is involved in the formation and maintenance of dendritic spines^54^,^55^ and is associated with autistic spectrum disorder. CTTNBP regulates dendritic arborization, a process that is particularly important in cerebellar Purkinje cells and that is progressively lost in A-T patients^56^. It is also important to note that neurogenesis and cell migration are two important processes during nervous system development that continue in adults. If ATM is, indeed, involved in the regulation of these neurodevelopmental processes, defects in ATM could explain the neurodegeneration observed in A-T patients, a hypothesis that awaits experimental validation.

## Methods

Species-specific static database searches for SCDs (defined as the presence of 3 S/TQ motifs in a stretch no longer than 100 amino acids) were performed using *SCDFinder* (http://ustbioinfo.webfactional.com/scd/). Results were filtered for the presence of multiple isoforms and multiple SCDs within a single protein were registered as single hits. *Homologene* (http://www.ncbi.nlm.nih.gov/homologene) was used for homology comparison across proteomes and *GOSTAT* (http://gostat.wehi.edu.au) was used to identify over- and under-represented ontologies in this list utilizing false discovery rate (Benjamin) to correct for multiple testing.

To test for the significance of the overlap between our 842 SCD-containing protein group and the a list of 686 human proteins containing phosphorylated S/TQ motifs identified in the large-scale proteomic analysis by Matsuoka *et al.*, a hypergeometric test was performed using the following parameters: number of draws (n) = 842, number of success (k) = 115, population size (N) = 33,972 and population successes (K) = 686. Same analysis was performed to test the significance of the overlap between our 842 SCD-containing protein group and 81 of the well-know ATM/ATR targets using the following parameters: number of draws (n) = 842, number of success (k) = 15, population size (N) = 33,972 and population successes (K) = 81. (Calculations done using the online calculator at https://www.geneprof.org/GeneProf/tools/hypergeometric.jsp).

## Additional Information

**How to cite this article**: Cara, L. *et al.* The ATM- and ATR-related SCD domain is over-represented in proteins involved in nervous system development. *Sci. Rep.* **6**, 19050; doi: 10.1038/srep19050 (2016).

## Supplementary Material

Supplementary Information
